# Exploring the association between social isolation and utilization of primary health services by older adults: evidence from China

**DOI:** 10.3389/fpubh.2024.1341304

**Published:** 2024-03-18

**Authors:** Xinlong Xie, Yanxia Lyu, Xinyu Li, Zhiruo Zhuang, Aijun Xu

**Affiliations:** ^1^School of Health Economics and Management, Nanjing University of Chinese Medicine, Nanjing, China; ^2^Jiangsu Research Center for Major Health Risk Management and TCM Control Policy, Nanjing University of Chinese Medicine, Nanjing, China

**Keywords:** social isolation, utilization of primary health services, propensity score matching (PSM), Chinese older adults, family isolation, friend isolation, community isolation

## Abstract

**Objective:**

This study aims to investigate the impact of social isolation on the utilization of primary health services among older adults in China.

**Methods:**

Data from the China Longitudinal Aging Social Survey (CLASS) conducted in 2018 were utilized. A binary logistic regression model was established, and propensity score matching (PSM) was employed for analysis.

**Results:**

The results of the binary logistic regression showed that family isolation within social isolation had a significant negative impact on the utilization of primary health services for older adults. In contrast, there was no significant association between friend isolation, community isolation, and the utilization of primary health services. Furthermore, the PSM results, using three matching methods (nearest neighbor matching, radius matching, and kernel matching), confirmed that family isolation significantly reduced older adults’ utilization of primary health services, consistent with the baseline regression findings.

**Conclusion:**

Reducing the occurrence of family isolation among older adults may be a cost-effective intervention measure. Efforts should be directed toward improving family support for older adults, promoting the utilization of primary health services, and strengthening disease prevention.

## 1 Introduction

Globally, populations are aging at an unprecedented rate. World Health Organization statistics (1) project that by 2050, the proportion of individuals aged 60 and above will increase from 12 to 22%. This demographic transition presents considerable challenges for public health systems, notably in the wake of the COVID-19 pandemic. The shift indicates not only a growing demand for healthcare services but also underscores the social participation challenges faced by older adults, with social isolation being a particular concern.

Social isolation is characterized by individuals having poor-quality social relationships or a scarcity of social networks across various levels of interaction (2). With age, people may become increasingly detached from their social networks due to the loss of work roles and changes in physical health, resulting in a state of limited communication and positioning them at a heightened risk for social isolation (3, 4). Research has consistently shown that social isolation adversely affects older adults’ physical activity (3, 5), mental health (6, 7), cognitive function (8, 9), cardiovascular health (10, 11), nutritional status (12), and sleep patterns (13, 14), and can even elevate the risk of mortality (15, 16), significantly impairing their quality of life (17). The social distancing measures implemented in response to COVID-19 have further amplified concerns regarding social isolation and its detrimental health outcomes (18, 19).

Primary healthcare institutions serve as essential gatekeepers for the health of urban and rural populations, playing a pivotal role in disease prevention, health counseling and education, as well as in providing care and rehabilitation services. Social isolation, as a critical social determinant, can hinder older adults’ access to medical care by limiting their information sources, increasing the difficulty of obtaining practical support, and diminishing their sense of belonging within social roles (20–22). It is, therefore, imperative to examine the relationship between social isolation and the utilization of primary healthcare services to enhance service accessibility for older adults.

While existing research has investigated the link between social isolation and health service utilization, findings remain inconclusive. Some studies report that socially isolated older adults are less likely to use outpatient and home medical care services (23–25), whereas others suggest no correlation or even a positive association between social isolation and healthcare utilization (26–29). Additionally, most studies inadequately assess the dimensions of social isolation, often relying on single scales or metrics to evaluate social connections and interaction frequency (30), without specifically addressing the critical types of social isolation—family, friends, and community—particularly within the context of collectivist cultures like China’s.

Accordingly, this paper examines the informal social support network in Chinese society, investigating how family, friend, and community isolation among older adults influences their utilization of primary healthcare services. This approach aims to deepen our understanding of the issue, offer targeted interventions for social isolation across different dimensions, and more effectively facilitate early disease detection, treatment, and rehabilitation. At a broader level, it seeks to address the challenges of population aging and promote healthy aging more effectively.

## 2 Methods

### 2.1 Data source and sample selection

This study utilizes data from the China Longitudinal Aging Social Survey (CLASS) conducted in 2018. CLASS is a nationwide, ongoing, large-scale social survey project initiated and implemented by the Survey and Data Center of Renmin University of China. The survey employed a stratified multistage probability sampling method, encompassing 28 provinces, cities, and autonomous regions across the country, ensuring a highly representative sample of the target population: individuals aged 60 and above. The comprehensive questionnaire included various domains, including basic demographic characteristics, health status, socioeconomic status, social support, psychological well-being, and family information, fulfilling the research requirements of this study. The initial sample size of CLASS in 2018 was 11,419. After excluding samples with missing values, the final sample size consisted of 8,343 older adults.

### 2.2 Measurement

#### 2.2.1 Utilization of primary health services

The dependent variable in this study is the utilization of primary health services among older adults. The questionnaire included a list of seven services: home care, home medical visits, rehabilitation training, rental of rehabilitation aids, physical examinations, establishment of health records, and attendance at health lectures. If older adults utilized any of these services, they were considered to utilize primary health services and were coded as 1. Otherwise, they were coded as 0.

#### 2.2.2 Social isolation

The independent variable in this study is social isolation, encompassing family isolation, friend isolation, and community isolation. The Lubben Social Network Scale (LSNS-6) is utilized in its abbreviated form to measure the levels of family and friend isolation among older adults (31). Family isolation (Cronbach’s alpha = 0.819) is assessed through three questions: “(1) How many family members/relatives do you meet or contact at least once a month? (2) How many family members/relatives can you have meaningful conversations with regarding personal matters? (3) How many family members/relatives can provide assistance when needed?” Similarly, friend isolation (Cronbach’s alpha = 0.843) is evaluated using the same three questions, with friends as the reference instead of family members. Respondents select from the following options for each question: 0 (none), 1 (1 person), 2 (2 persons), 3 (3–4 persons), 4 (5–8 persons), and 5 (9 or more persons). If the total score for family or friend isolation falls below 6 (18), it is considered as to indicate the presence of family or friend isolation and is classified as 1. Conversely, a score of 6 or higher, indicates the absence of family or friend isolation and is coded as 0.

Community isolation is assessed through the following question: “In the past 3 months, have you participated in any of the following activities? Such as community security patrols, caregiving for other older adults, environmental hygiene protection, mediation of disputes, socializing with companions, providing voluntary services with specialized skills, assisting in the care of other people’s children, etc.” If respondents have not participated in any of the mentioned activities, it is considered to indicate the presence of community isolation and coded as 1. Conversely, participation in any of these activities is coded as 0, indicating the absence of community isolation among older adults.

#### 2.2.3 Covariates

In this study, covariates were selected based on the Andersen Healthcare Utilization Model, considering three dimensions: predisposing characteristics, enabling resources, and health needs (32). The specific covariates are as follows: (1) Predisposing characteristics encompass age (>60 years), gender (male, female), education level (illiterate, primary school, middle school, high school, or above), marital status (married, unmarried/divorced/widowed), and household registration (rural, non-rural). (2) Enabling resources consist of income (logarithm of personal income in the past year), pension insurance (presence of at least one type of pension insurance, none), employment status (employed, unemployed), number of living children (continuous variable), and child caregiving support (1. almost daily, 2. at least once a week, 3. at least once a month, 4. a few times a year, and 5. almost never). (3) Health needs include chronic illness (presence of at least one chronic illness, none), self-rated health assessment (1. very unhealthy, 2. somewhat unhealthy, 3. average, 4. somewhat healthy, and 5. very healthy), and cognitive ability.

Cognitive ability is evaluated using the Mini-Mental State Examination (MMSE) Folstein et al. (33). The 2018 CLASS survey questionnaire incorporated items related to time orientation, place orientation, immediate memory, delayed memory, attention, and calculation. Scores on the MMSE range from 0 to 16, with lower scores indicating poorer cognitive function.

### 2.3 Statistical methods

The dependent variable in this study is the “utilization of primary health services,” which is represented as a binary dummy variable. Therefore, a binary logistic regression model is employed to examine the impact of social isolation on the usage of primary health services among older adults. The specific model is specified as follows:

$$\ln\left[\frac{P\left(D_{i}=1\right)}{1-P\left(D_{i}=1\right)}\right]=\alpha_{0}+\overset{3}{\underset{k=1}{\sum }}\alpha_{k}X_{ki}+\alpha_{4}\mathrm{Contro}l_{i}+\mu_{i}$$

Where *D_i_* = 1 indicates that older adult *i* utilizes primary health services, while *D_i_* = 0 indicates non-utilization. *X_ki_* represents the social isolation variables related to the *k*-th dimension that may affect the utilization of primary health services among older adults. *Control* represents the covariates, *μ* represents the random disturbance term, *i* denotes the individual-level observation, and *α* represents the estimated parameter values.

Social isolation, an outcome of various factors in older adults, may introduce endogeneity due to selection bias. In this study, a counterfactual framework is employed, and the propensity score matching (PSM) method is used to mitigate selection bias and confounding bias, as well as to robustly test the baseline regression results (34, 35). The PSM method reduces the multidimensionality of the compared groups to a one-dimensional propensity score and matches cases with similar scores using various matching algorithms. Under the assumptions of balance check and common support, the average treatment effect (ATT) for the treated group is calculated using the following expression:

$$ATT_{PSM}=E\left(y_{1i}-y_{0i}\;|\;X_{i}=1\right)=E\left(y_{li}\;|\;X_{i}=1\right)-E\left(y_{0i}\;|\;X_{i}=1\right)$$

Where *X_i_* = 1 indicates that older adult *i* is in a social isolation state, *X_i_* = 0 indicates that older adult *i* is not in a social isolation state, *y_1i_* represents the utilization of primary health services for older adults in a social isolation state, and *y_0i_* represents the utilization of primary health services for older adults not in a social isolation state. *ATT_PSM_* represents the difference between the utilization of primary health services for older adults in a social isolation state, *E*(*y_1i_|X_i_ =* 1), and the utilization of primary health services for older adults not in a social isolation state, *E*(*y_0i_|X*i *=* 1). All statistical analyses in this study were conducted using STATA 17.0.

## 3 Results

### 3.1 Characteristics of study participants

Table 1 displays the social isolation status of older adults. Among the 8,343 participants, 25.28% experienced family isolation, 36.65% experienced friend isolation, and 65.55% experienced community isolation. The risk of experiencing these isolations increased with a higher level of relationship distance. Importantly, in this study, the chi-square test was utilized and revealed significant differences only in family isolation (*p* < 0.001) between older adults who utilized primary healthcare services and those who did not.

**Table 1:** Statistical description of analysis samples created from CLASS 2018.

Characteristic	Total sample	Utilization of primary healthcare services		*χ*^2^/*t*	*p*
		Use	No use		
Observations *n* (%)	8,343	2,763 (33.12)	5,580 (66.88)		
Family isolation *n* (%)					
Isolated	2,109 (25.28)	614 (22.22)	1,495 (26.79)	20.43	0.000
Not isolated	6,234 (74.72)	2,149 (77.78)	4,085 (73.21)		
Friend isolation *n* (%)					
Isolated	3,058 (36.65)	1,016 (36.77)	2,042 (36.59)	0.03	0.875
Not isolated	5,285 (63.35)	1,747 (63.23)	3,538 (63.41)		
Community isolation *n* (%)					
Isolated	5,469 (65.55)	1,805 (65.33)	3,664 (65.66)	0.09	0.761
Not isolated	2,874 (34.45)	958 (34.67)	1,916 (34.34)		
Age mean ± SD	71.29 ± 7.29	72.08 ± 7.46	70.90 ± 7.18	−6.86	0.000
Gender *n* (%)					
Male	4,197 (50.31)	1,321 (47.81)	2,876 (51.54)	10.29	0.001
Female	4,146 (49.69)	1,442 (52.19)	2,704 (48.46)		
					
Education level *n* (%)					
Illiterate	1,984 (23.78)	727 (26.31)	1,257 (22.53)	26.53	0.000
Primary school	3,321 (39.81)	1,128 (40.83)	2,193 (39.30)		
Junior high school	2,084 (24.98)	624 (22.58)	1,460 (26.16)		
High school or above	954 (11.43)	284 (10.28)	670 (12.01)		
Marital status *n* (%)					
Married	5,872 (70.38)	1,895 (68.58)	3,977 (71.27)	6.40	0.011
Single ^a^	2,471 (29.62)	868 (31.42)	1,603 (28.73)		
Household registration *n* (%)					
Rural	4,116 (49.33)	1,237 (44.77)	2,879 (51.59)	34.44	0.000
Non-rural	4,227 (50.67)	1,526 (55.23)	2,701 (48.41)		
Lnincome mean ± SD	8.24 ± 1.44	8.38 ± 1.41	8.18 ± 1.44	−6.02	0.000
Pension insurance *n* (%)					
Yes	6,599 (79.10)	2,463 (89.14)	4,136 (74.12)	252.16	0.000
No	1,744 (20.90)	300 (10.86)	1,444 (25.88)		
Employment status *n* (%)					
Employed	2,002 (24.00)	546 (19.76)	1,456 (26.09)	40.63	0.000
Unemployed	6,341 (76.00)	2,217 (80.24)	4,124 (73.91)		
Number of living children mean ± SD	2.55 ± 1.32	2.48 ± 1.31	2.58 ± 1.32	3.31	0.001
Child caregiving support *n* (%)					
Almost daily	1,711 (20.51)	628 (22.73)	1,083 (19.41)	55.73	0.000
At least once a week	2,387 (28.61)	878 (31.78)	1,509 (27.04)		
At least once a month	1,474 (17.67)	474 (17.16)	1,000 (17.92)		
A few times a year	1,090 (13.06)	295 (10.68)	795 (14.25)		
Almost none	1,681 (20.15)	488 (17.66)	1,193 (21.38)		
Chronic disease *n* (%)					
Yes	6,375 (76.41)	2,209 (79.95)	4,166 (74.66)	28.69	0.000
No	1,968 (23.59)	554 (20.05)	1,414 (25.34)		
Self-rated health mean ± SD	3.36 ± 0.86	3.42 ± 0.86	3.33 ± 0.86	−4.35	0.000
Cognitive ability mean ± SD	13.46 ± 3.12	12.84 ± 3.41	13.77 ± 2.91	12.22	0.000

^a^Single: Not married/divorced/widowed; SD, Standard deviation.

Table 1 also presents the characteristics of older adults and their utilization of primary health services. Among the participants, 50.31% were male, 39.81% had completed primary education, and 70.38% were married. Approximately 76.41% had at least one chronic illness. The average age of the participants was 71.29 ± 7.29 years. The mean logarithm of income was 8.24 ± 1.44. They had an average of 2.55 ± 1.32 living children, with an average caregiving support score from children of 2.84 ± 1.42. The average self-rated health status was 3.36 ± 0.86, and the mean cognitive ability score was 13.46 ± 3.12. Furthermore, significant differences at the 0.1 level were found between older adults who utilized primary health services and those who did not, based on chi-square tests/t-tests. These differences pertained to age, gender, educational level, marital status, household registration, income, pension insurance, employment status, number of living children, caregiving support from children, chronic illness, self-rated health, and cognitive ability.

### 3.2 Impact of social isolation on the utilization of primary health services among older adults

In this study, the utilization of primary health services was considered as the dependent variable, while family isolation, friend isolation, and community isolation were treated as independent variables. Covariates with a significance level of *p* < 0.1 in the univariate analysis were included in the binary logistic regression model for further analysis. The results are presented in Table 2. Models 1–1 to 1–3 in the table, respectively, demonstrate the impact of family isolation, friend isolation, and community isolation on the utilization of primary health services among older adults. The results indicate that out of the three dimensions of social isolation, only family isolation significantly reduced the utilization of primary health services (OR = 0.855; 95% CI: 0.763 ~ 0.958; *p* < 0.01). However, there was no significant association observed between friend isolation, community isolation, and the utilization of primary health services.

**Table 2:** Regression coefficient and odds ratios (95% confidence intervals) of the impact of social isolation on the utilization of primary health services among older adults, CLASS 2018.

Characteristic	Model I		Model II		Model III	
	Coefficient	OR (95% CI)	Coefficient	OR (95% CI)	Coefficient	OR (95% CI)
Family isolation	−0.156^***^	0.855 (0.763–0.958)				
Friend isolation			0.047	1.049 (0.948–1.160)		
Community isolation					0.016	1.016 (0.917–1.126)
Gender	0.054	1.056 (0.955–1.166)	0.059	1.061 (0.960–1.172)	0.058	1.06 (0.959–1.171)
Age	0.015^***^	1.015 (1.007–1.023)	0.014^***^	1.014 (1.006–1.022)	0.014^***^	1.014 (1.006–1.022)
Education level	−0.208^***^	0.812 (0.765–0.862)	−0.203^***^	0.816 (0.769–0.866)	−0.204^***^	0.815 (0.768–0.865)
Marital status	−0.081	0.922 (0.822–1.035)	−0.085	0.919 (0.819–1.031)	−0.084	0.919 (0.819–1.031)
Household registration	0.099	1.104 (0.976–1.249)	0.097	1.101 (0.974–1.246)	0.098	1.102 (0.975–1.247)
Lnincome	0.044^**^	1.045 (1.004–1.088)	0.05^**^	1.051 (1.010–1.094)	0.049^**^	1.05 (1.009–1.094)
Pension insurance	1.037^***^	2.82 (2.445–3.252)	1.057^***^	2.876 (2.494–3.318)	1.049^***^	2.856 (2.476–3.293)
Number of living children	−0.129^***^	0.879 (0.842–0.917)	−0.123^***^	0.884 (0.847–0.922)	−0.125^***^	0.883 (0.846–0.921)
Employment status	−0.148^**^	0.862 (0.758–0.981)	−0.141^**^	0.868 (0.763–0.987)	−0.144^**^	0.866 (0.761–0.985)
Child caregiving support	−0.068^***^	0.934 (0.902–0.968)	−0.073^***^	0.93 (0.898–0.963)	−0.072^***^	0.93 (0.899–0.963)
Self-rated health	0.233^***^	1.262 (1.190–1.339)	0.235^***^	1.265 (1.193–1.342)	0.235^***^	1.265 (1.192–1.341)
Chronic disease	0.403^***^	1.496 (1.328–1.685)	0.399^***^	1.491 (1.324–1.679)	0.399^***^	1.49 (1.322–1.679)
Cognitive ability	−0.101^***^	0.904 (0.889–0.919)	−0.101^***^	0.904 (0.889–0.919)	−0.102^***^	0.903 (0.889–0.918)
Constant	−1.694^***^	0.184 (0.087–0.390)	−1.784^***^	0.168 (0.079–0.357)	−1.753^***^	0.SSSS (0.082–0.367)
Number of observations	8,343		8,343		8,343	
Pseudo *r*-squared	0.064		0.064		0.064	

OR, Odds ratio; CI, Confidence intervals. ^∗^, ^∗∗^, and ^∗∗∗^ indicate significance at the levels of 10, 5, and 1%, respectively.

The analysis of the impact of family isolation is reported in Table 2. Age, education level, income, pension insurance, number of surviving children, employment status, caregiving support from children, self-rated health, chronic illness, and cognitive function showed significant effects on the utilization of primary health services among older adults, with significance observed at the 0.1 level. It is noteworthy that higher education level (OR = 0.812; 95% CI: 0.765–0.862; *p* < 0.01), a greater number of surviving children (OR = 0.879; 95% CI: 0.842–0.917; *p* < 0.01), employment status (OR = 0.862; 95% CI: 0.758–0.981; *p* < 0.05), less caregiving support from children (OR = 0.934; 95% CI: 0.902–0.968; *p* < 0.01), and better cognitive ability (OR = 0.904; 95% CI: 0.889–0.919; *p* < 0.01) were identified as risk factors for older adults not utilizing primary health services.

### 3.3 Robustness check

Family isolation among older adults can be influenced by various factors, potentially introducing endogeneity issues due to sample selection bias. To address these concerns and ensure the robustness of the baseline regression results, this study incorporates a comprehensive set of covariates (34) in the research model and employs three matching methods: nearest neighbor matching (*K* = 1), radius matching (Cal = 0.01), and kernel matching.

#### 3.3.1 Balance check

**Figure 1:**
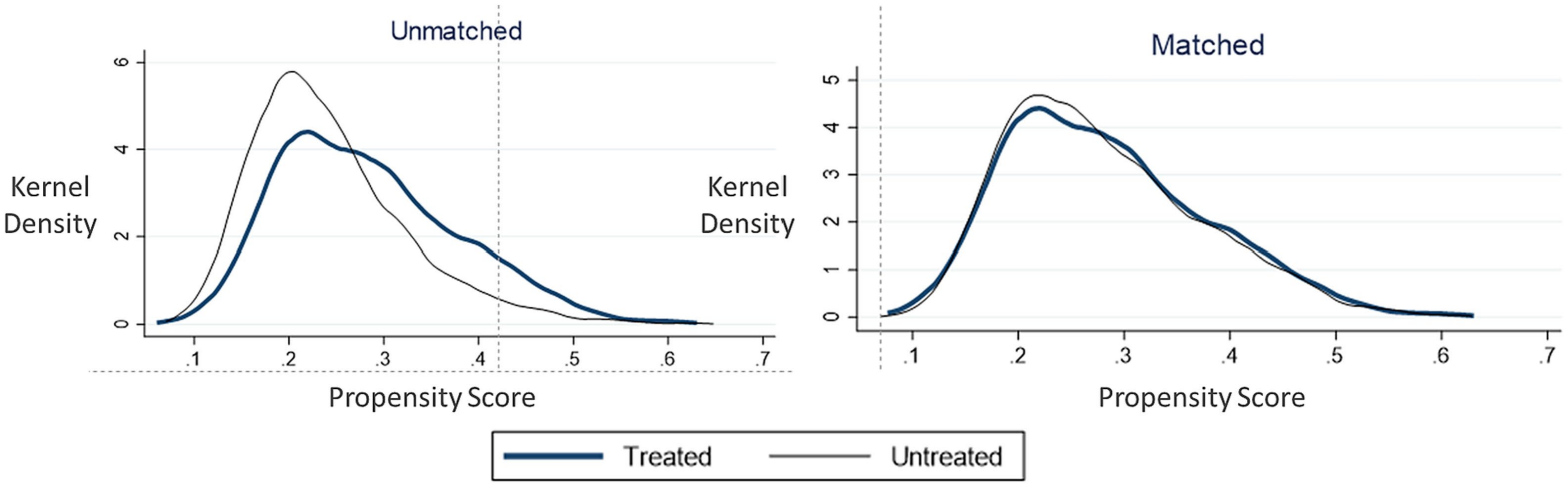
Kernel density distribution plot before and after kernel matching.

To ensure matching quality, we estimated propensity scores using a logit model, and conducted balance checks to assess the similarity of the treatment and control groups in key characteristics. Table 3 shows that the standard errors for all covariates decrease after matching, with absolute values below10%. Additionally, the t-test results show significant reductions in differences between the experimental and control groups, indicating a partial resolution of sample heterogeneity. Table 4 demonstrates a substantial decrease in Pseudo R^2^ values, implying a reduction in the explanatory power of matching variables for family isolation among older adults after matching. The post-matching data aligns more closely with the assumption of conditional randomness. Furthermore, the mean bias and med bias values are below 5%, indicating a high level of consistency in the matching characteristics. Figure 1 displays the density distribution of propensity values after kernel matching, demonstrating improved balance and matching results compared to the pre-matching distribution.

**Table 3:** The situation of error reduction before and after variable matching.

Variable	Unmatched	Mean		% bias	%reduct|bias|	*t*-test	
	Matched	Treated	Control			*t*	*p* > t
Gender	U	1.4808	1.5024	−4.3		−1.72	0.086
	M	1.4806	1.4824	−0.4	91.2	−0.12	0.902
Age	U	71.565	71.199	5		1.99	0.046
	M	71.559	71.514	0.6	87.7	0.2	0.842
Education level	U	2.1366	2.2759	−14.8		−5.88	0
	M	2.1357	2.1458	−1.1	92.7	−0.35	0.723
Marital status	U	1.3139	1.2902	5.2		2.06	0.039
	M	1.314	1.3126	0.3	94	0.1	0.921
Household registration	U	0.46894	0.51941	−10.1		−4.01	0
	M	0.46869	0.47148	−0.6	94.5	−0.18	0.856
Lnincome	U	7.9583	8.3406	−27		−10.64	0
	M	7.9571	7.9864	−2.1	92.3	−0.67	0.505
Pension insurance	U	0.71313	0.81729	−24.8		−10.23	0
	M	0.713	0.7213	−2	92	−0.6	0.55
Number of living children	U	2.4609	2.5752	−8.7		−3.44	0.001
	M	2.4578	2.4653	−0.6	93.4	−0.19	0.847
Employment status	U	0.24514	0.23821	1.6		0.64	0.52
	M	0.24526	0.24445	0.2	88.3	0.06	0.951
Child caregiving support	U	3.0915	2.7514	23.8		9.56	0
	M	3.092	3.0688	1.6	93.2	0.52	0.601
Self-rated health	U	3.3011	3.3774	−8.9		−3.53	0
	M	3.3008	3.3055	−0.6	93.8	−0.18	0.858
Chronic disease	U	0.78615	0.75666	7		2.76	0.006
	M	0.78605	0.782	1	86.2	0.32	0.749
Cognitive ability	U	13.545	13.436	3.5		1.39	0.165
	M	13.544	13.525	0.6	82.3	0.2	0.839

**Table 4:** Test of matching balance values.

Method	Sample	Ps *R*^2^	LR	*p*	Mean bias	Med bias
Nearest neighbor matching (*K* = 1)	Unmatched	0.035	330.90	0.00	11.10	8.70
	Matched	0.001	5.77	0.95	1.80	1.40
Radius matching (Cal = 0.01)	Unmatched	0.035	330.90	0.00	11.10	8.70
	Matched	0.000	0.26	1.00	0.40	0.40
Kernel matching	Unmatched	0.035	330.90	0.00	11.10	8.70
	Matched	0.000	1.40	1.00	0.90	0.60

Ps, Pseudo; LR, Likelihood ratio.

#### 3.3.2 Common support test

**Figure 2:**
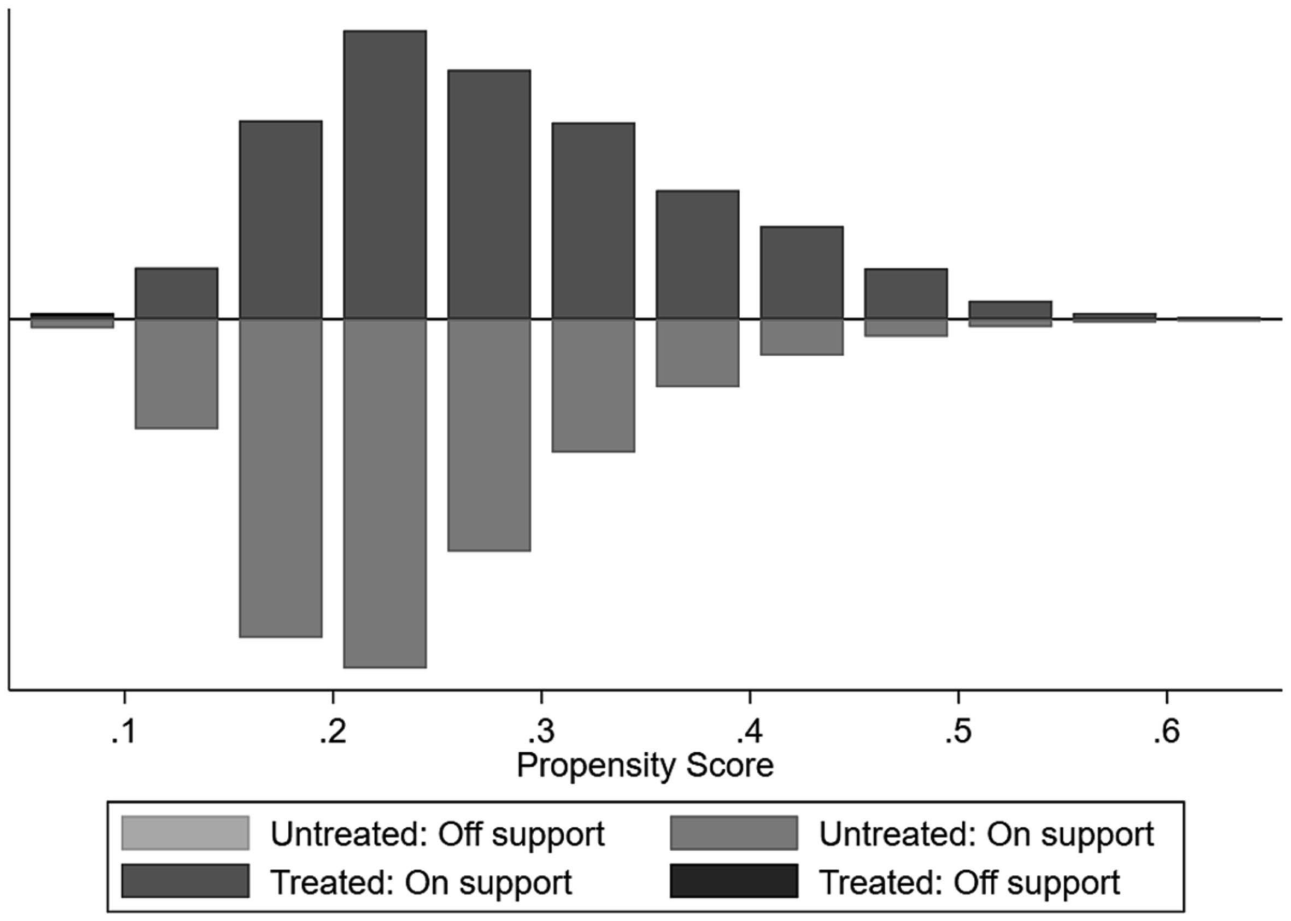
Test of common support.

Following matching, it is crucial to evaluate the distribution’s consistency and substantially reduced biases between the treatment and control groups, as well as determine the degree to which these groups are comparable. Figure 2 displays the distribution of propensity scores for both groups and illustrates the region of common support. The results reveal that approximately 99% of the matched samples fall within the region of common support, providing support for the assumption of common support. Only samples falling within the region of common support are utilized in the subsequent analysis.

#### 3.3.3 Estimation of the impact of family isolation on the utilization of primary health services among older adults

Table 5 presents the average treatment effect on the treated (ATT) for family isolation, as obtained from the three matching methods: −0.036, −0.024, and − 0.026, respectively. All ATT values are statistically significant at the 0.05 level, implying a substantial reduction in the likelihood of older adults experiencing family isolation utilizing primary health services. These findings corroborate with the baseline regression results, solidifying the robustness of the study’s conclusions.

**Table 5:** The average treatment effect of propensity score matching among three matching methods.

Method	Treated	Controls	ATT	S.E	T-stat
Nearest neighbor matching (*K* = 1)	0.291	0.327	−0.036^**^	0.017	−2.15
Radius matching (Cal = 0.01)	0.291	0.315	−0.024^**^	0.012	−2.01
Kernel matching	0.291	0.318	−0.026^**^	0.012	−2.21

S.E., Standard error; ATT, The average treatment effect on the treated. ^*^, ^**^, and ^***^ indicate significance at the levels of 10, 5, and 1%, respectively.

## 4 Discussion

This study used the 2018 CLASS data and employed statistical tools like binary logistic regression and (PSM). Building on the relational sequences of informal social support in China, the study investigated the influence of social isolation on the utilization of primary health services by older adults across the three dimensions of family isolation, friend isolation, and community isolation. This broadened our comprehension of the use of primary health services patterns with an aging population. Social isolation is an important public health concern, and the study distinguished between its dimensions, enhancing the systematic analysis of social isolation and contributing new micro-level data for examining its effect on the utilization of primary health services by older adults.

The study found that the occurrence rates of family isolation, friend isolation, and community isolation among older adults were 25.28, 36.65, and 65.55%, respectively, with family isolation exhibiting a comparatively lower occurrence rate. However, based on the Andersen model, the results revealed that only family isolation had a significant negative impact on the utilization of primary health services among older adults, while friend isolation and community isolation demonstrated no significant association with the utilization of primary health services.

Currently, consensus on the relationship between social isolation and health service utilization among older adults has not been achieved in existing literature. Some scholars argue for a negative correlation between social isolation and health service utilization (23, 25, 36), aligning with the results of this study regarding family isolation. Specifically, it has been observed that socially isolated older adults exhibit lower utilization of outpatient and primary care services, but a higher likelihood of future hospitalizations and emergency room visits (23, 37). These findings may be attributed to the crucial role of family in providing emotional and material support (38, 39), which effectively alleviates healthcare burdens faced by older adults (27, 38) and assists in the identification of healthcare needs, including preventive measures (38). Moreover, due to limitations in behavior and emotional well-being, socially isolated older adults may face challenges in effectively managing their own health (40). In the absence of informal support, there may be delays in receiving care, leading to reduced utilization of primary health services. Additionally, studies have confirmed that improving relationships with family members and relatives contributes to increased utilization of health services and preventive care among older adults, relieving psychological and financial pressures encountered in the healthcare process (24, 41). Conversely, socially isolated and lonely older adults encounter difficulties in social engagement, which hinders their access to actual support (21) and diminishes their sense of social role significance (22), ultimately leading to reduced utilization of health services by older adults (23, 42). Furthermore, social isolation has been associated with cognitive decline and a decreased likelihood of seeking help (43, 44). This could be due to communication challenges faced by socially isolated older adults in effectively interacting with healthcare professionals, leading them to seek assistance primarily from family members who are more knowledgeable about their health conditions. Consequently, when faced with primary healthcare issues, older adults may prefer seeking support from their families rather than relying on friends and community resources, as family members possess a better understanding of their health and can provide more convenient healthcare assistance.

Of course, there is ongoing debate among scholars regarding the relationship between social isolation and the utilization of health services among older adults. Some argue for a significant positive correlation (28, 29, 45–47), while others find no significant association (26, 37), which is somewhat inconsistent with the findings of this study. These discrepancies may be attributed to social isolation being recognized as a major public health concern (28, 46–49), leading older adults to seek health services support fulfill their social needs. and interact with healthcare providers. However, risk factors associated with social isolation, such as declining health and functional decline (45, 50–52), are common reasons for older adults to utilize health services (53–56). Some researchers have found that after adjusting for baseline demographic characteristics and pre-existing chronic conditions, social isolation is no longer significantly associated with most types of health service utilization (26, 45, 57), which is consistent with the findings of this study regarding friend isolation and community isolation. Additionally, there have been studies reporting a reversal in the relationship between social isolation and planned outpatient treatments (58, 59). It is worth considering that this study focuses on objective social isolation, which refer s to the actual lack of social connections rather than subjective perceptions of isolation (60), unlike previous studies that have predominantly focused on perceived isolation (28, 45, 46, 61). Additionally, it has been suggested that health service utilization should be differentiated between planned medical care and emergency medical care (23, 37), with the utilization examined in this study leaning more towards preventive planned care, while hospitalization and emergency services incline more towards emergency care. Therefore, differences in results among scholars may be stemming from distinctions between objective social isolation and subjective perceived isolation, as well as the differentiation of planned and emergency health service utilization.

Furthermore, our research findings revealed a significant correlation between lower levels of support from adult children and a decrease in the utilization of primary health services among older adults, thus partially supporting the hypothesis that family isolation contributes to a reduction in primary health service utilization (38, 39, 41). Surprisingly, having a larger number of living adult children was also identified as a risk factor for lower utilization of primary health services. This finding can be explained by the fact that having more adult children not only provides enhanced support in seeking medical care but also leads to heightened attention to the health status of older adults in their daily lives (62), resulting in a substitution effect on primary health service utilization (63, 64). However, our study also revealed a contrary finding that higher levels of cognitive ability were associated with lower utilization of primary health services, contradicting to our initial hypothesis (43, 44). This could be attributed to the fact that cognitive decline increases health risks (65, 66), thereby leading to higher utilization of primary health services among older adults.

Considering the detrimental effects of family isolation on older adults’ health and well-being, this study presents a suite of evidence-based strategies designed to alleviate its impact through comprehensive interventions and to offer effective support to older adults lacking family support. The proposed strategies include: Firstly, designing and implementing initiatives by policymakers and social service organizations to bolster older adults’ social engagement through community activities and interest groups, thereby enhancing their social connections and community integration. Secondly, enhancing family caregivers’ capabilities and motivation through targeted training and support services, thereby improving the quality of care for older adults. For those living alone or without family support, creating a volunteer network to facilitate regular home visits and companionship services could significantly diminish feelings of isolation and marginalization. Lastly, leveraging modern information technology, such as smart home systems and remote health monitoring, could help maintain older adults’ connections with the external world and bolster their independence.

This study offers valuable insights into how social isolation affects older adults’ use of primary health services, though it also presents certain limitations. Firstly, the reliance on self-reported questionnaires for data collection, while practical and efficient for broad data gathering, inherently carries biases due to memory recall, comprehension differences, and response inclinations, potentially compromising the data’s objectivity and accuracy. Consequently, the study’s conclusions should be considered in light of these potential biases in self-reporting. Future research could benefit from employing a mix of data collection methods, including qualitative interviews alongside quantitative data, to achieve a deeper, more nuanced understanding. Secondly, the data for this study was gathered before the COVID-19 pandemic, a period that significantly altered patterns of social isolation and health service use among the global older adults. The lockdown might have provided family members with more opportunities to be with older adults, possibly alleviating some aspects of social isolation. However, the pandemic’s stressors, mobility restrictions, and health anxieties could have intensified social isolation, adversely affecting their patterns of using primary health services. These specific contextual factors should be taken into account when interpreting this study’s findings, facilitating a more comprehensive grasp of the complex ways in which social isolation influences health service use among older adults. Future studies should aim to collect and analyze data during and post-COVID-19 pandemic to evaluate the pandemic’s lasting effects on social isolation among older adults and their use of primary health services. Lastly, the cross-sectional design of this study restricts our capacity to establish causal relationships between the variables. Since data in cross-sectional studies are captured at a single point in time, it remains uncertain whether family isolation directly results in decreased use of primary health services among older adults. Therefore, the study cannot conclusively state that family isolation is a direct cause for the reduced use of primary health services by older adults. To gain a clearer understanding of the causal links among these variables, future research should employ a longitudinal design, examining the causal dynamics over time through participant data tracking, thus providing a firmer scientific foundation for developing targeted interventions.

## 5 Conclusion

In conclusion, our study findings demonstrate a significant negative association between family isolation and primary health services utilization among older adults, while friend isolation and community isolation do not exhibit a significant relationship. This underscores the importance of addressing family isolation as a potentially cost-effective intervention to promote primary health service utilization and strengthen disease prevention efforts in this growing demographic.

## Data availability statement

The original contributions presented in the study are included in the article/supplementary material, further inquiries can be directed to the corresponding author.

## Ethics statement

Ethical review and approval were not required for the study on human participants in accordance with the local legislation and institutional requirements. The patients/participants provided their written informed consent to participate in this study. The studies were conducted in accordance with the local legislation and institutional requirements.

## Author contributions

XX: Writing – original draft. YL: Writing – review & editing. XL: Writing – original draft, Writing – review & editing. ZZ: Writing – review & editing. AX: Writing – original draft, Writing – review & editing.
